# Independent and joint associations of hypertension and depression with cardiovascular diseases and all-cause mortality: a population-based cohort study

**DOI:** 10.1038/s41371-025-01045-1

**Published:** 2025-07-17

**Authors:** Qiang Tu, Shuanglan Lin, Nashid Hafiz, Karice Hyun, Deborah Manandi, Emma Zhao, Haisheng Wu, Yangxi Huang, Shuzhen Ma, Zhengqiu Zhang, Jiazhen Zheng, Julie Redfern

**Affiliations:** 1 Faculty of Medicine and Health, The University of Sydney, Camperdown, NSW Australia; 2 School of Nursing, Dali University, Dali, China; 3 Department of Cardiology, Concord Hospital, Sydney, NSW Australia; 4 School of Public Health, The University of Hong Kong, Hong Kong, China; 5 School of Nursing, The University of Hong Kong, Hong Kong, China; 6 School of Public Administration, Guilin University of Technology, Guilin, China; 7 School of Physical Education and Sports, Soochow University, Suzhou, China; 8 Bioscience and Biomedical Engineering Thrust, Systems Hub, The Hong Kong University of Science and Technology (Guangzhou), Guangzhou, Guangdong China

**Keywords:** Risk factors, Hypertension

## Abstract

Hypertension frequently co-exists with depression, leading to adverse health outcomes. This study aimed to examine the individual and joint effects of hypertension and depression on the risks of new-onset cardiovascular disease (CVD) and all-cause mortality among the middle-aged and older Chinese individuals. Data from the China Health and Retirement Longitudinal Study (CHARLS) during 2011–2020 were used. Participants were divided into four groups for comparison: hypertension alone, depression alone, both conditions, neither condition. Multivariate logistic regression models were established to compare the risks of all-cause mortality and CVD among the four groups. A total of 9178 participants without pre-existing CVD were included and followed for nine years. Compared with individuals with neither condition, the risk of all-cause mortality increased among individuals with hypertension alone (adjusted odds ratio [aOR]: 1.414, 95% confidence interval [CI]: 1.133–1.764), depression alone (aOR: 1.023, 95% CI: 0.795–1.317) and comorbid hypertension and depression (aOR: 1.524, 95% CI: 1.180–1.968). The aORs for CVD events in individuals with both conditions, hypertension alone, and depression only were 2.207 (95% CI: 1.885–2.584), 1.945 (95% CI: 1.702–2.222) and 1.572 (95% CI: 1.365–1.809), respectively. Furthermore, those with severe depressive symptoms were at higher risks of all-cause mortality and CVD, regardless of having hypertension. Hypertension with comorbid depression leads to higher risks of CVD and all-cause mortality than either condition alone. Screening and management of depression among individuals with hypertension are essential for the primary prevention of CVD and premature death.

## Introduction

Hypertension is the leading cause of cardiovascular disease (CVD) and premature death worldwide [1]. It affects one in three adults worldwide and accounts for 13% of the total of all deaths [2]. The global prevalence of hypertension is increasing significantly. According to the report of World Health Organisation, the number of adults living with hypertension doubled from 650 million in 1990 to 1.3 billion in 2019 [2]. In accordance with the global trend, the prevalence of hypertension in China increased from 5% in 1959 to 37% in 2017, making China has the largest population with hypertension (approximately 270 million) in the world [1, 3]. The rapid increase in hypertension prevalence places enormous disease and societal economic burden globally and in China [4].

Depression is a common mental disorder, which frequently co-exists with hypertension. There is evidence of a bidirectional relationship between hypertension and depression due to shared biological pathways [5]. The prevalence of comorbid depression among patients with hypertension has been reported to be as high as 27% [6]. A meta-analysis of prospective cohort studies revealed that depressive patients had 1.4-fold greater risk of having hypertension [7]. Previous population-based studies have found that hypertension and depression are both independent predictors of CVD and mortality [8, 9]. Importantly, comorbid depression in patients with hypertension is clinically significant because of its strong association with suboptimal compliance to healthy lifestyles and medication treatment [10]. There is compelling evidence that hypertension combined with depression is related to adverse outcomes in terms of suboptimal blood pressure control [11], lower quality of life [12] and increased use of healthcare resources [5, 6].

A few cohort studies have been conducted to assess the joint effects of hypertension and depression on the risks of CVD [13–15] and mortality [13, 16–18]. However, existing studies are all based on samples from western countries [13–17] and are limited by small sample size [13], enrolment of participants many decades ago [16–18], or restriction to selected patient population rather than general population [13, 14]. It is worth noting that majority of the previous studies [13, 14, 16, 17, 19] incorporated patients with previous diagnosed CVD and focused on the evaluation of re-occurrence of CVD. Therefore, the association of hypertension and depression with new-onset CVD is still unclear. To date, no prospective population-based studies in China have examined the individual and joint associations of hypertension and depression with the risks of new-onset CVD and mortality. Therefore, the aim of the current study was to address the contextual gap and to evaluate the individual and joint effects of hypertension and depression on the risk of new-onset CVD and all-cause mortality among the middle-aged and elderly Chinese population.

## Methods

### Study design and participants

This study was a secondary analysis of the data from the China Health and Retirement Longitudinal Study (CHARLS), which is an ongoing nationwide cohort study among the middle-aged and elderly populations from 150 counties and 450 communities across 28 provinces in China [20]. CHARLS has collected a wide range of socioeconomic and health data using a structured questionnaire via face-to-face interviews. A total of 17708 participants were recruited in the baseline survey occurred in 2011 (wave 1). Additional four waves of follow-up surveys were respectively conducted in 2013 (wave 2), 2015 (wave 3), 2018 (wave 4) and 2020 (wave 5). In the present study, 9-year follow-up data from 2011–2020 were retrospectively analysed. Ethics approval for CHARLS was granted by the Ethics Review Committee of Peking University (IRB00001052-11015). Details of the study design and sampling methods of CHARLS is published elsewhere [20].

**Fig. 1:**
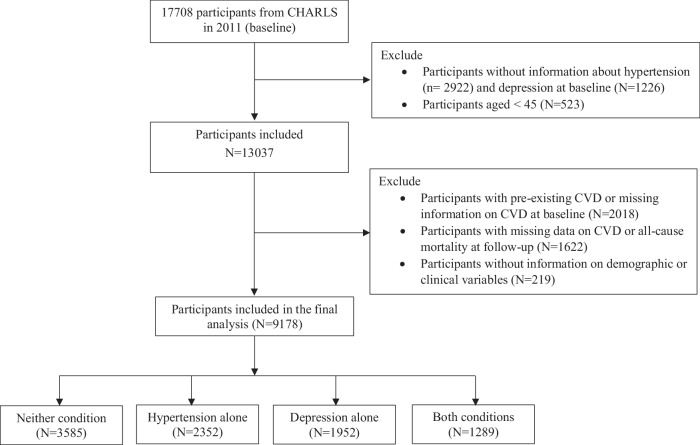
Flow chart of sample selection.

In the current study, participants who met all the following criteria were included: (1) without history of CVD at baseline; (2) aged ≥ 45 years; (3) had information about hypertension and depression at baseline; (4) without missing data and lost from follow-up. Finally, a sample of 9178 middle-aged and older Chinese adults without CVD at baseline were included for subsequent analysis. A flow chart of the sample selection process is presented in Fig. 1.

### Assessment of depression and hypertension

Depression was identified using the 10-item Centre for Epidemiological Studies Depression Scale (CESD-10). The Chinese version of the CESD-10 has demonstrated high reliability and validity and has been used as a screening tool for depression in the community residents [21]. CESD-10 consists of 10 items assessing different dimensions of depressive symptoms: (1) bothered by little things, (2) had trouble concentrating, (3) felt depressed, (4) everything was an effort, (5) felt hopeful, (6) felt fearful, (7) sleep was restless, (8) felt happy, (9) felt lonely, and (10) could not get going. Participants rated the frequency of each symptom on a 4-point Likert scale. The total score ranged from 0–30, with a higher score reflecting more severe depressive symptoms. A cut-off score of ≥10 was used to identify individuals with depression [22]. In addition, participants with antidepressant use at baseline were identified as having depression.

Hypertension was ascertained based on physical measurement and self-reporting. In accordance with the diagnostic criteria of American Heart Association [23], hypertension was defined as systolic blood pressure (SBP) ≥ 140 mmHg and/or diastolic blood pressure (DBP) ≥ 90 mmHg and/or current use of antihypertensive medications and/or self-reported history of hypertension. For example, participants were identified as having hypertension if they had a previous diagnosis of hypertension or were on antihypertensive medications, even when their blood pressure readings were lower (i.e., SBP < 140 mmHg and/or DBP < 90 mmHg). Furthermore, we also identified hypertensive individuals whose blood pressure readings exceeded the normal range (i.e., systolic blood pressure ≥140 mmHg and/or diastolic blood pressure ≥90 mmHg) but were not on antihypertensive medications and did not have a prior history of hypertension. Blood pressure was measured three times using an electronic sphygmomanometer (Omron HEM-7200 Monitor). The mean value of three measurements was taken as the final blood pressure of each participant.

### Study outcomes

The primary outcomes were new-onset CVD events and all-cause mortality from 2011–2020. The secondary outcomes were new-onset stroke and cardiac events during the 9-year follow-up. In common with previous studies [24, 25], incident CVD events were ascertained based on self-reported physician diagnosis (“Have you been told by a doctor that you have been diagnosed with a heart attack, angina, coronary heart disease, heart failure, or other heart problems?” or “Have you been told by a doctor that you have been diagnosed with a stroke?”). Participants who reported heart disease or stroke during the follow-up period were defined as new-onset CVD patients.

### Covariates

The covariates collected at baseline included gender, age, marital status (married vs. other marital status [never married, separated, divorced or widowed]), residence status (rural vs. urban), highest educational level (below elementary school, elementary school, middle school, high school or above), smoking status (ever vs. never smoking), drinking status (ever vs. never drinking), body mass index (BMI), clinical outcomes (systolic blood pressure [SBP], diastolic blood pressure [DBP]), and chronic comorbidities (dyslipidemia, chronic lung disease, liver disease, kidney disease, stomach or other digestive disease, arthritis or rheumatism, and asthma).

### Statistical analyses

Participants were divided into four mutually exclusive groups based on their status of hypertension and depression: (i) neither hypertension nor depression, (ii) depression alone, (iii) hypertension alone, and (iv) comorbid hypertension and depression. Characteristics of participants at baseline were presented as mean ± standard deviation (SD) for normally distributed continuous data, or median (interquartile range, IQR) for continuous data with a skewed distribution. Categorical variables were expressed as frequencies and percentages. Comparisons were made using Chi-square tests, ANOVA or Kruskal-Wallis test as appropriate.

Binary logistic regression models were performed to assess the prospective association of hypertension and depression with all-cause mortality, stroke, cardiac events, and CVD. Three models were established: (1) Model 1 was a crude model without any adjustment; (2) Model 2 was adjusted for age and gender; (3) Model 3 was multivariable adjusted, including age, gender, education, marital status, residence, BMI, drinking status, smoking status, and chronic comorbidities. The results were presented as odds ratio (OR) and 95% confidence interval (95% CI). In all models, the group of participants with neither hypertension nor depression was treated as the reference group. We also examined potential interactions between depression and hypertension on additive and multiplicative scales. The additive interactions were measured using the relative excess risk due to interaction (RERI), attribution proportion due to interaction (AP), and synergy index (SI). RERI indicates the excess risk due to the interaction relative to the risk without exposure. RERI = OR_hypertension and depression_-OR_hypertension_-OR_depression_+1. AP is interpreted as the proportion of the risk that is due to interaction among individuals with both exposures. AP=RERI/OR_hypertension and depression_. SI represents the excess risk from both exposures when there is interaction relative to the risk from exposure without interaction. SI = [OR_hypertension and depression_-1]/[(OR_hypertension_-1)+(OR_depression_-1)] [26]. If the 95% CIs of RERI and AP did not include 0 and the 95% CI of SI did not include 1, there is an additive interaction between the two risk factors [27]. Multiplicative interactions were assessed by adding a product term of depression and hypertension to the models. In addition, we divided depression into three levels (CESD-10 score: <10, 10–19 and ≥20) to evaluate the impact of different levels of depression on the risks of CVD and all-cause mortality.

Subgroup analyses were conducted to examine the potential modification by sex (male vs. female) and age (middle-aged vs. older) in the risk estimates. Furthermore, two sensitivity analyses were conducted to test the robustness of the results. First, we repeated the main analysis by using a more stringent cutoff (CESD-10 ≥ 12) to define depression. It was reported that a CESD-10 score ≥12 exhibited a higher sensitivity in identifying clinically depressive symptoms [28]. Second, we further adjusted antidepressant medications in the multivariable models. The statistical analyses were performed using Stata 18.0 (StataCorp., College Station, TX). A two-sided P value of <0.05 was considered statistically significant.

## Results

### Baseline characteristics

Of the 9178 participants included for analysis, 48.7% were male and the mean (SD) age was 58.11 (8.94) years. Table 1 summarises the baseline characteristics of the participants by hypertension and depression. In total, 3585 (39.0%) had neither condition, 2352 (25.6%) had hypertension alone, 1952 (21.2%) had depression alone, and 1289 (14.0%) had both conditions. Significant differences among the four groups were found for all covariates. Compared to participants with neither condition, those with comorbid hypertension and depression were more likely to be women, older, unmarried, live in rural area, have higher BMI, have lower level of education, and have higher prevalence of chronic comorbidities.

**Table 1:** Baseline characteristics of the participants.

Characteristic	Neither hypertension nor depression	Hypertension alone	Depression alone	Both hypertension and depression	Overall	*p* value
	(*n* = 3585)	(*n* = 2352)	(*n* = 1952)	(*n* = 1289)	(*n* = 9178)	
Men, *n* (%)	1953 (54.5%)	1227 (52.2%)	800 (41.0%)	486 (37.7%)	4466 (48.7%)	<0.001
Age (years), mean (SD)	55.98 (8.14)	60.11 (9.20)	57.38 (8.57)	61.50 (9.36)	58.11 (8.94)	<0.001
Married, n (%)	3336 (93.1%)	2080 (88.4%)	1721 (88.2%)	1017 (78.9%)	8154 (88.8%)	<0.001
Rural residence, *n* (%)	2328 (64.9%)	1376 (58.5%)	1462 (74.9%)	903 (70.1%)	6069 (66.1%)	<0.001
Smoking, *n* (%)	1546 (43.1%)	975 (41.5%)	698 (35.8%)	451 (35.0%)	3670 (40.0%)	<0.001
Drinking, *n* (%)	1490 (41.6%)	1004 (42.7%)	696 (35.7%)	484 (37.5%)	3674 (40.0%)	<0.001
BMI (kg/m^2^), mean (SD)	23.00 (3.42)	24.60 (4.34)	22.33 (3.50)	23.87 (4.16)	23.36 (3.87)	<0.001
BMI category, *n* (%)						
Underweight	211 (5.9%)	86 (4.0%)	202 (10.5%)	79 (6.8%)	578 (6.6%)	<0.001
Normal weight	2466 (69.4%)	1164 (54.4%)	1353 (70.4%)	665 (57.0%)	5648 (64.3%)	
Overweight or obesity	877 (24.7%)	889 (41.6%)	366 (19.1%)	423 (36.2%)	2555 (29.1%)	
Education, *n* (%)						
Primary school or lower	2174 (60.6%)	1555 (66.1%)	1459 (74.7%)	1038 (80.5%)	6226 (67.8%)	<0.001
Middle school	901 (25.1%)	494 (21.0%)	362 (18.5%)	187 (14.5%)	1944 (21.2%)	
High school or above	510 (14.2%)	303 (12.9%)	131 (6.7%)	64 (5.0%)	1008 (11.0%)	
CES-D-10 score	4.36 (2.80)	4.32 (2.71)	15.01 (4.42)	15.16 (4.48)	8.13 (6.20)	<0.001
Clinical outcomes						
SBP (mm Hg), mean (SD)	118.70 (11.41)	148.45 (19.25)	117.41 (11.58)	147.34 (20.04)	129.36 (20.80)	<0.001
DBP (mm Hg), mean (SD)	71.11 (8.67)	84.32 (11.84)	69.65 (8.84)	83.34 (11.87)	75.59 (11.93)	<0.001
Chronic conditions						
Dyslipidemia, *n* (%)	158 (4.4%)	308 (13.1%)	93 (4.8%)	152 (11.8%)	711 (7.7%)	<0.001
Chronic lung disease, n (%)	227 (6.3%)	188 (8.0%)	202 (10.3%)	143 (11.1%)	760 (8.3%)	<0.001
Liver disease, *n* (%)	85 (2.4%)	61 (2.6%)	80 (4.1%)	35 (2.7%)	261 (2.8%)	0.002
Kidney disease, *n* (%)	126 (3.5%)	87 (3.7%)	129 (6.6%)	81 (6.3%)	423 (4.6%)	<0.001
Digestive disease, *n* (%)	663 (18.5%)	312 (13.3%)	571 (29.3%)	329 (25.5%)	1875 (20.4%)	<0.001
Arthritis, *n* (%)	879 (24.5%)	629 (26.7%)	819 (42.0%)	570 (44.2%)	2897 (31.6%)	<0.001
Asthma, *n* (%)	86 (2.4%)	76 (3.2%)	104 (5.3%)	79 (6.1%)	345 (3.8%)	<0.001

*BMI* body mass index, *DBP* diastolic blood pressure, *SBP* systolic blood pressure, *SD* standard deviation.

### Independent and joint associations of depression and hypertension with all-cause mortality and CVD

Over the nine-year follow-up, a total of 2381 (25.9%) participants experienced new-onset CVD events (830 [9.0%] stroke and 1821 [19.8%] cardiac events) and 729 (7.9%) participants died. As shown in Table 2, hypertension or depression was independently associated with an increased risk of CVD in all models. In addition, hypertension alone increased the risk of all-cause mortality. Importantly, the risks of all-cause mortality and CVD were highest in participants with both conditions. After adjustment for confounding factors, the risk estimates were slightly diminished, but remained highest among the four groups.

**Table 2:** Association of hypertension and depression with all-cause mortality and CVD.

Variables	Neither hypertension nor depression	Hypertension alone	Depression alone	Both hypertension and depression	Overall
	(*n* = 3585)	(*n* = 2352)	(*n* = 1952)	(*n* = 1289)	(*n* = 9178)
All-cause mortality					
Case, *n*(%)	192 (5.4%)	247 (10.5%)	126 (6.5%)	164 (12.7%)	729 (7.9%)
Unadjusted	1.000 (Ref)	2.074 (1.704–2.523)^***^	1.219 (0.967–1.538)	2.576 (2.069–3.207)^***^	
Age and sex-adjusted	1.000 (Ref)	1.404 (1.138–1.732)^**^	1.148 (0.898–1.468)	1.712 (1.348–2.176)^***^	
Multivariable-adjusted^a^	1.000 (Ref)	1.414 (1.133–1.764)^**^	1.023 (0.795–1.317)	1.524 (1.180–1.968)^**^	
CVD^b^					
Case, *n*(%)	604 (16.8%)	768 (32.7%)	505 (25.9%)	504 (39.1%)	2381 (25.9%)
Unadjusted	1.000 (Ref)	2.393 (2.116–2.706)^***^	1.722 (1.507–1.969)^***^	3.169 (2.749–3.652)^***^	
Age and sex-adjusted	1.000 (Ref)	2.223 (1.962–2.519)^***^	1.639 (1.432–1.876)^***^	2.797 (2.417–3.237)^***^	
Multivariable-adjusted^b^	1.000 (Ref)	1.945 (1.702–2.222)^***^	1.572 (1.365–1.809)^***^	2.207 (1.885–2.584)^***^	
Stroke					
Case, *n*(%)	158 (4.4%)	313 (13.3%)	168 (8.6%)	191 (14.8%)	830 (9.0%)
Unadjusted	1.000 (Ref)	3.330 (2.729–4.063)^***^	2.043 (1.632–2.557)^***^	3.773 (3.023–4.708)^***^	
Age and sex-adjusted	1.000 (Ref)	3.155 (2.578–3.860)^***^	2.055 (1.639–2.576)^***^	3.603 (2.869–4.523)^***^	
Multivariable-adjusted^b^	1.000 (Ref)	2.674 (2.161–3.309)^***^	2.058 (1.631–2.597)^***^	2.824 (2.210–3.609)^***^	
Cardiac events^c^					
Case, *n*(%)	483 (13.5%)	552 (23.5%)	389 (19.9%)	397 (30.8%)	1821 (19.8%)
Unadjusted	1.000 (Ref)	1.970 (1.720–2.255)^***^	1.598 (1.380–1.851)^***^	2.858 (2.455–3.328)^***^	
Age and sex-adjusted	1.000 (Ref)	1.836 (1.600–2.108)^***^	1.494 (1.289–1.732)^***^	2.486 (2.125–2.909)^***^	
Multivariable-adjusted^a^	1.000 (Ref)	1.622 (1.399–1.879)^***^	1.402 (1.201–1.636)^***^	2.005 (1.692–2.376)^***^	

^**^p < 0.01, ^***^p < 0.001.

^a^Multivariable-adjusted for age, gender, education, marital status, residence, BMI, drinking, smoking, and chronic comorbidities.

^b^CVD including stroke and cardiac events.

^c^Cardiac events included myocardial infarction, coronary heart disease, angina, congestive heart failure, or other heart problems.

Compared with those with neither condition, the multivariable-adjusted ORs for all-cause mortality were 1.414 (95% CI 1.133–1.764) for individuals with hypertension alone, 1.023 (95% CI 0.795–1.317) for individuals with depression alone, and 1.524 (95% CI 1.180–1.968) for individuals with both conditions, respectively. Compared with those with neither condition, the multivariable-adjusted risk of CVD was 94.5% higher among individuals with hypertension alone (aOR 1.945, 95% CI 1.702–2.222), 57.2% higher among individuals with depression alone (aOR 1.572, 95% CI 1.365–1.809) and 120.7% higher among individuals with both conditions (aOR 2.207, 95% CI 1.885–2.584). In addition, individuals with both conditions also showed highest odds of incident stroke (aOR 2.824, 95% CI 2.210–3.609) and cardiac events (aOR 2.005, 95% CI 1.692–2.376) among the four groups. To explore the additive interaction of depression and hypertension on all-cause mortality and CVD, RERI, AP and SI were calculated (Table 3). After adjusting for potential confounders, they did not show any significant additive interaction (RERI_all-cause mortality_=0.087, 95% CI −0.346–0.519, and RERI_CVD_=−0.309, 95% CI −0.691–0.072, respectively). Furthermore, we did not observe any multiplicative interactions (p for multiplicative interaction >0.05).

**Table 3:** Additive interaction between hypertension and depression on all-cause mortality and CVD.

Outcomes	Additive interaction			Multiplicative interaction (95% CI)
	RERI (95% CI)	AP (95% CI)	SI (95% CI)	
All-cause mortality	0.087 (−0.346–0.519)	0.056 (−0.223–0.336)	1.198 (0.461–3.111)	1.053 (0.745–1.487)
CVD	−0.309 (−0.691–0.072)	−0.140 (−0.322–0.424)	0.795 (0.607–1.052)	0.722 (0.588–0.886)
Stroke	−0.908 (−1.648–0.166)	−0.321 (−0.608–0.034)	0.667 (0.485–0.918)	0.882 (0.705–1.102)
Cardiac events	−0.019 (−0.380–0.343)	−0.009 (−0.190–0.171)	0.981 (0.687–1.401)	0.513 (0.376–0.700)

Multivariable-adjusted for age, gender, education, marital status, residence, BMI, drinking, smoking, and chronic comorbidities.

*AP* attributable proportion. It indicates the proportion of the risk that is due to interaction among individuals with both exposures. AP = RERI/OR _hypertension and depression_. *RERI* relative excess risk for interaction. It indicates the excess risk due to the interaction relative to the risk without exposure. RERI = OR _hypertension and_ _depression_ - OR _hypertension_ - OR _depression_+1. *SI* synergy index. It represents the excess risk from both exposures when there is interaction relative to the risk from exposure without interaction. SI = [OR _hypertension and depression_ -1]/[(OR _hypertension_ - 1)+(OR _depression_ - 1)].

**Fig. 2:**
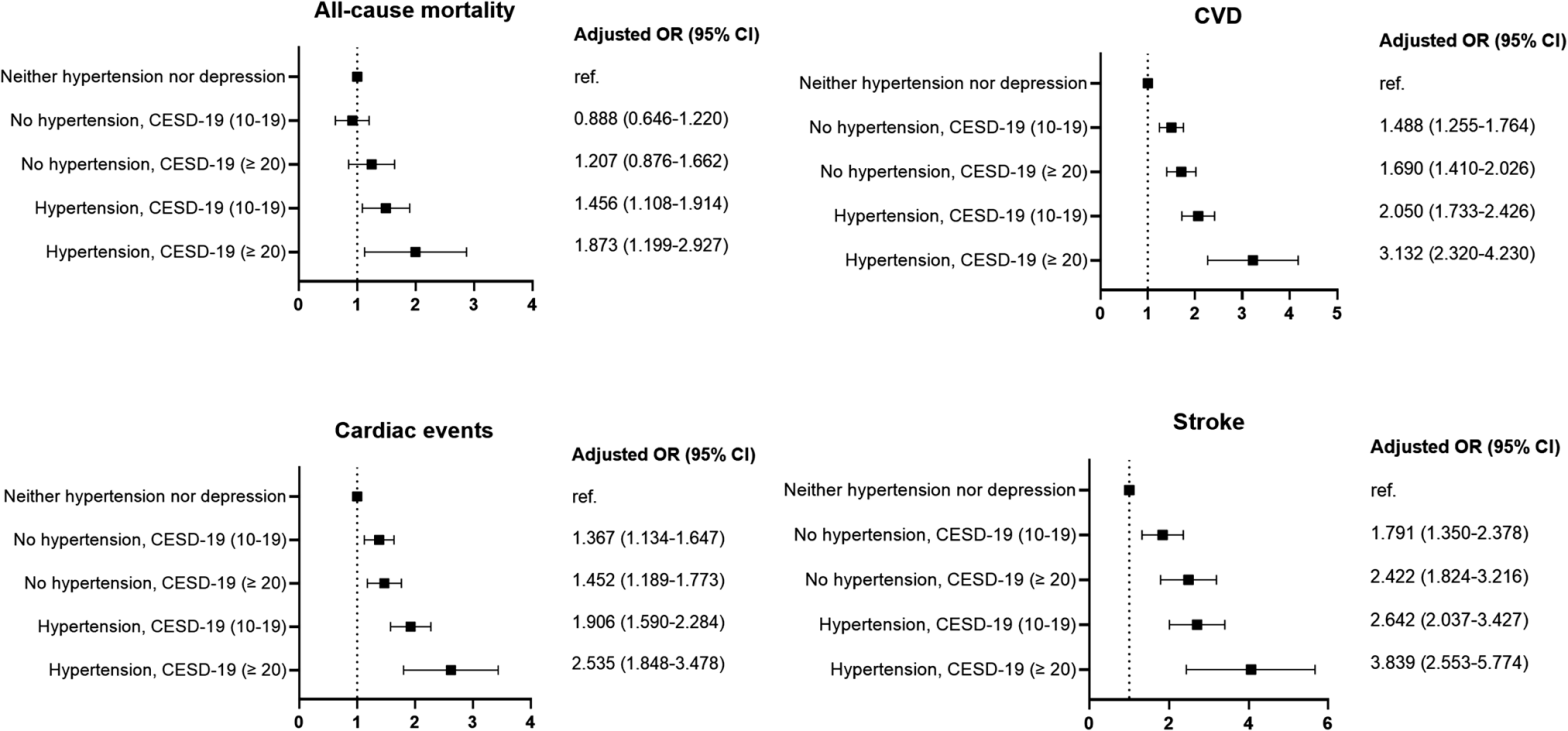
The impact of different levels of depression on the risks of all-cause mortality, CVD, stroke and cardiac events.

In Fig. 2, we present the impact of different levels of depression on the risks of CVD and all-cause mortality. In the full-adjusted models, we found that the risks of all-cause mortality and CVD increased along with elevated depressive symptoms, regardless of having hypertension or not. The adjusted risk estimates of CVD (aOR 3.132, 95% CI 2.320–4.230) and all-cause mortality (aOR 1.873, 95% CI 1.199–2.927) were highest in hypertensive patients with severe depression (CESD-10 ≥ 20).

### Subgroup analyses and sensitivity analyses

In the subgroup analyses, the risks of CVD and all-cause mortality remained highest among those with both conditions, independent of age and sex (Table 4). The association of comorbid hypertension and depression with CVD appeared to be more pronounced among male (aOR 2.541, 95% CI 1.985–3.254) and middle-aged adults (aOR 2.276, 95% CI 1.887–2.745) compared to their counterparts. The risk of all-cause mortality was higher among those with both conditions who were female (aOR 1.883, 95% CI 1.223–2.899) and younger than 65 years (aOR 1.581, 95% CI 1.064–2.349). In the sensitivity analyses, the main results remained robust when a higher cut-off value of 12 on the CESD-10 scale was used to define depression (Supplementary Table 1). When antidepressant medications were further adjusted in the multivariable models, results were similar to the main analyses (Supplementary Table 2).

**Table 4:** Multivariable-adjusted odds ratio stratified by sex and age.

Outcomes	Characteristics	Neither hypertension nor depression	Hypertension alone	Depression alone	Both hypertension and depression
All-cause mortality	Sex				
	Male	1.000 (Ref)	1.386 (1.066–1.802)^*^	0.858 (0.625–1.177)	1.454 (1.011–2.197)^*^
	Female	1.000 (Ref)	1.539 (1.007–2.351)^*^	1.473 (0.946–2.294)	1.883 (1.223–2.899)^**^
	Age				
	< 65	1.000 (Ref)	1.548 (1.122–2.137)^**^	0.980 (0.683–1.407)	1.581 (1.064–2.349)^*^
	≥ 65	1.000 (Ref)	1.334 (0.978–1.820)	1.082 (0.754–1.554)	1.478 (1.046–2.090)^*^
CVD	Sex				
	Male	1.000 (Ref)	2.304 (1.909–2.781)^***^	1.664 (1.341–2.064)^***^	2.541 (1.985–3.254)^***^
	Female	1.000 (Ref)	1.636 (1.353–1.980)^***^	1.472 (1.221–1.775)^***^	1.968 (1.600–2.421)^***^
	Age				
	< 65	1.000 (Ref)	2.009 (1.723–2.343)^***^	1.478 (1.261–1.733)^***^	2.276 (1.887–2.745)^***^
	≥ 65	1.000 (Ref)	1.778 (1.347–2.347)^***^	1.892 (1.381–2.591)^***^	2.111 (1.552–2.872)^***^
Stroke	Sex				
	Male	1.000 (Ref)	2.941 (2.226–3.886)^***^	1.832 (1.312–2.558)^***^	2.940 (2.064–4.189)^***^
	Female	1.000 (Ref)	2.376 (1.702–3.316)^***^	2.218 (1.587–3.099)^***^	2.673 (1.883–3.794)^***^
	Age				
	< 65	1.000 (Ref)	2.822 (2.210–3.603)^***^	2.062 (1.584–2.684)^***^	2.961 (2.217–3.953)^***^
	≥ 65	1.000 (Ref)	2.201 (1.412–3.429)^***^	2.007 (1.209–3.332)^**^	2.412 (1.490–3.903)^***^
Cardiac events	Sex				
	Male	1.000 (Ref)	1.976 (1.594–2.451)^***^	1.483 (1.158–1.897)^**^	2.317 (1.760–3.050)^***^
	Female	1.000 (Ref)	1.361 (1.110–1.668)^**^	1.327 (1.087–1.620)^**^	1.816 (1.460–2.259)^***^
	Age				
	< 65	1.000 (Ref)	1.693 (1.428–2.006)^***^	1.309 (1.098–1.560)^**^	2.041 (1.668–2.499)^***^
	≥ 65	1.000 (Ref)	1.440 (1.062–1.952)^*^	1.700 (1.210–2.389)^**^	1.957 (1.406–2.725)^***^

Multivariable-adjusted for age, gender, education, marital status, residence, BMI, drinking, smoking, and chronic comorbidities.

^*^p < 0.05, ^**^p < 0.01; ^***^p < 0.001.

## Discussion

This is the first study to examine the longitudinal association of hypertension and depression with first-episode CVD and all-cause mortality among the Chinese population. The present study provided real-world evidence that hypertension and depression were both independently associated with increased risk of CVD. The simultaneous presence of hypertension and depression conferred greater risks of CVD and all-cause mortality than either condition. This association remained significant after adjustment for potential confounding variables. Consistent results were shown in the subgroup analyses and sensitivity analyses, demonstrating the robustness of our findings.

Compared to participants with neither condition, those with comorbid hypertension and depression were more likely to have higher prevalence of chronic comorbidities such as hyperlipidemia. Furthermore, hyperlipidemia was more prevalent among those with hypertension than those with depression. Epidemiological studies showed that hyperlipidemia is frequently coexisted with hypertension due to the similar pathophysiological etiologies such as obesity and artery narrowing [29, 30]. In addition, patients with hypertension are prone to insulin resistance, which hinders the breakdown of lipids and leads to hyperlipidemia [29]. On the other hand, hyperlipidemia may facilitate the development of hypertension by negatively affecting the functional and structural properties of arteries. These changes may compromise blood pressure regulation, thereby increasing the risks of developing hypertension among individuals with hyperlipidemia [30].

There is considerable evidence supporting that hypertension and depression were independent risk factors of CVD. A meta-analysis of 18 longitudinal studies revealed that depression was associated with a 1.7-fold increased risk of CVD [31]. Another meta-analysis of 41 studies confirmed that hypertension was an established risk factor of CVD and found that a significant dose-response relationship between SBP levels and risk of ischemic heart disease [32]. The individual effect of hypertension and depression shown in our study is consistent with most of the existing evidence. However, an analysis of UK Biobank data found that the risk of stroke was not significantly increased among patients with hypertension alone or depression alone [15]. Furthermore, non-significant association was observed for depression incident cardiovascular events in their study. These conflicting results might be attributed to the sample characteristics distinctions, disparities in health service level between countries, divergent response to the disease, or residual confounding effects.

Consistent with the previous studies [13, 14], we found that comorbid depression was associated with a greater risk of CVD among patients with hypertension. A cohort study conducted in general practices found that depression significantly increased the risk of coronary events by 18% among elderly patients with hypertension in the Netherlands [13]. However, their study was limited by a relatively small sample with only 555 participants, indicating a possibility of insufficient statistical power in the examination of study hypothesis. In another UK-based study consisting of 2656 hypertensive patients above 80 years with 2 years followed-up, it was found that the risks of incident stroke and cardiovascular events in patients with depression were 1.8 and 1.6 times higher than those without depression, respectively [14]. However, their study did not exclude participants with pre-existing CVD at baseline. Therefore, it was possible that depression was resulted from CVD, indicating their results might subject to reverse causation. Together, these two studies only restricted study participants to those with hypertension and were not able to assess whether the presence of hypertension modified the excess risk of CVD associated with depression. Therefore, our study extended their study by identifying that the presence of comorbid hypertension might exacerbate the effects of depression as a risk factor for CVD.

Findings of the present study on mortality are similar to a previous observational study in UK which reported that individuals with both hypertension and mental disorders had a higher risk of all-cause mortality than those with neither condition [17]. However, their study focused on patients with mental disorders incorporating anxiety and depression and did not specifically evaluate the influence of depression. Furthermore, Axon et al. analysed data on 10025 participants from the National Health and Nutrition Epidemiologic Follow-up Study I (NHANES I) and found that participants with both hypertension and depression were associated with 1.4-fold greater risk of all-cause mortality after 8 years of follow-up compared with those with neither condition [16]. However, their interview follow-up occurred during 1971–1975 and the threshold for diagnosing hypertension (SBP/DBP > 160/95 mmHg) was different with the current diagnosis criteria (SBP/DBP > 140/90 mmHg). Therefore, their study may have underestimated the risk difference between groups as patients with hypertension might have been included in the no hypertension group.

The higher risks of mortality and CVD in patients with comorbid hypertension and depression could potentially be explained by behavioural (i.e. poor adherence to medical treatment) and biological (i.e. autonomic dysfunction and chronic inflammation) mechanisms [33]. Depression has been shown to adversely affect adherence to health behaviors and medication treatment [34]. Therefore, coexistence of these two condition may further complicate treatment, increase the risk of CVD and worsen physical health [10]. Furthermore, suicide resulting from depression could be another plausible explanation of increased mortality among patients with hypertension [35]. On the other hand, depression could induce chronic low-grade inflammation and trigger hyperactivity of hypothalamic-pituitary-adrenal axis, resulting in high levels of cortisol [36]. The effects of cortisol on both vasoconstriction and elevated blood pressure could lead to endothelial impairment and thrombus formation, ultimately accelerating the occurrence of CVD and CVD-related death of patients with hypertension [37].

Our results imply that special clinical attention should be paid to the coexistence of hypertension and depression. Given that depression is often underdiagnosed [38], it is essential to raise awareness of clinicians in appreciating the risk associated with depression among patients with hypertension. Efforts should focus on regular screening, early detection and effective management of depression among patients with hypertension in order to strengthen the primary prevention of CVD and premature death [33]. In addition, there may be potential benefits from close monitoring and proactive management of blood pressure in patients with depression. Importantly, implementation of integrated care strategies that simultaneously target hypertension and depression might be a promising approach to reduce the burden of CVD and mortality. A meta-analysis has provided convincing evidence supporting that combination treatment of depression and hypertension is beneficial for health outcomes [39]. However, well-designed clinical trials of examining the effectiveness of integrated care interventions that simultaneously targeting these two risk factors are required before its widespread adoption in clinical practices. In addition, it is crucial to conduct process evaluation to assess fidelity and acceptance of the interventions and identify the contextual factors that may impact intervention implementation.

The strengths of our study included a large, nationally representative cohort with a long follow-up period, as well as a specific focus on first-episode CVD events. However, the limitations of this study should be noted. First, depression diagnosis relied on a questionnaire and antidepressant use, rather than a formal clinical assessment. However, the CESD-10 is proved as a reliable and valid instrument that is widely used for depression screening in population studies [21]. Future studies using clinically diagnosed samples are needed to validate our findings. Second, diagnosis of CVD was self-reported, which may lead to some degree of recall bias. Third, due to the observational nature of the cohort studies, there is a possibility of residual confounding stemming from unmeasured confounding factors (e.g., income, social support etc.). As an observational study, it is difficult to establish a causal relationship between exposures and outcomes. Fourth, participants were classified based on their baseline exposure status. However, health status of participants might have changed during the follow-up period. For example, transition from persistent to temporary symptoms of depression in the follow-up period may affect the classification of participants and the study results. Fifth, the exact cause of death was not collected in the CHARLS database, making us unable to evaluate the impacts on cause-specific mortality (e.g., cardiac death). Furthermore, data on specific CVD events were not available. Due to the limitations of study surveys, we were unable to specify additional heart conditions in the cardiac event definition and identify the effects of hypertension and depression on the specific type of CVD events (e.g. heart failure, mitral regurgitation). Future studies are needed to evaluate the impacts of hypertension and depression on the specific CVD events. Sixth, we were unable to identify the individual effects of behavioural and biological mechanisms on the overall results based on the existing data. Future studies are needed to identify the individual impacts of behavioural and biological mechanisms on the overall effects. Seventh, information on the past clinical diagnosis of depression was unavailable in CHARLS. There is a possibility of misclassification of depression as we could not ascertain the history of depression diagnoses among participants. Finally, our study only included Chinese participants and so our findings may have limited generalisability to other ethnic groups.

## Conclusion

Our findings showed that presence of hypertension or depression significantly increased the risk of first-episode CVD. Comorbid hypertension and depression conferred greater risks of CVD and all-cause mortality than either condition alone. Furthermore, the risks of all-cause mortality and CVD increased along with elevated depressive symptoms, regardless of having hypertension or not. These findings highlight that early screening, detection and management of depression are important for the primary prevention of CVD and premature death, particularly for individuals with hypertension.

## Summary

### What is known about topic

- Hypertension is a significant risk factor of CVD.
- Depression is prevalent among patients with hypertension.
- There is a lack of studies examining the combined effects of hypertension and depression on the new-onset CVD.

### What this study adds

- Hypertension with comorbid depression is associated with greater risks of new-onset CVD and all-cause mortality than either condition alone.
- The risks of CVD and all-cause mortality increase along with the elevated depressive symptoms among individuals with hypertension.
- The study highlights the importance of depression screening and management among individuals with hypertension to prevent CVD and mortality.

## Supplementary information

Supplementary material 1

Supplementary material 2

## Data Availability

Data for this study are publicly available at the China Health and Retirement Longitudinal Study website (https://charls.pku.edu.cn/en/) upon reasonable request.
