# Supplementary material for: Independent and joint associations of hypertension and depression with cardiovascular diseases and all-cause mortality: a population-based cohort study
Source: J Hum Hypertens. 2025 Jul 17;39(9):634–42. doi: 10.1038/s41371-025-01045-1 (PMC12417204; doi:10.1038/s41371-025-01045-1)
Supplement: Supplementary file 1 — Supplementary material 1 [file 41371_2025_1045_MOESM1_ESM.docx]

**Supplementary table 1.** **Sensitivity analyses using a cut-off score of 12 for depression**

| Variables | Neither hypertension nor depression  (N=4098) | Hypertension alone  (N=2679) | Depression alone  (N=1439) | Both hypertension and depression  (N=962) | All participants  (n = 9178) |
| --- | --- | --- | --- | --- | --- |
| All-cause mortality |  |  |  |  |  |
| Case, n(%) | 225 (5.5%) | 278 (10.4%) | 93 (6.5%) | 133 (13.8%) | 729 (7.9%) |
| Unadjusted | 1.000 (Ref) | 1.993 (1.660-2.393)^***^ | 1.189 (0.927-1.526) | 2.762 (2.201-3.466)^***^ |  |
| Age and sex-adjusted | 1.000 (Ref) | 1.377 (1.131-1.675)^**^ | 1.146 (0.881-1.492) | 1.813 (1.413-2.327)^***^ |  |
| Multivarialbe-adjusted^†††^ | 1.000 (Ref) | 1.378 (1.120-1.695)^**^ | 0.999 (0.761-1.312) | 1.605 (1.229-2.097)^**^ |  |
| CVD† |  |  |  |  |  |
| Case, n(%) | 716 (17.5%) | 891 (33.3%) | 393 (27.3%) | 381 (39.6%) | 2381 (25.9%) |
| Unadjusted | 1.000 (Ref) | 2.354 (2.101-2.638)^***^ | 1.775 (1.541-2.044)^***^ | 3.097 (2.660-3.607)^***^ |  |
| Age and sex-adjusted | 1.000 (Ref) | 2.191 (1.951-2.460)^***^ | 1.684 (1.461-1.942)^***^ | 2.714 (2.320-3.173)^***^ |  |
| Multivarialbe-adjusted^†††^ | 1.000 (Ref) | 1.902 (1.679-2.154)^***^ | 1.611 (1.388-1.870)^***^ | 2.138 (1.804-2.534)^***^ |  |
| Stroke |  |  |  |  |  |
| Case, n(%) | 195 (4.8%) | 364 (13.6%) | 131 (9.1%) | 140 (14.6%) | 830 (9.0%) |
| Unadjusted | 1.000 (Ref) | 3.147 (2.625-3.773)^***^ | 2.005 (1.593-2.523)^***^ | 3.409 (2.709-4.290)^***^ |  |
| Age and sex-adjusted | 1.000 (Ref) | 2.984 (2.482-3.588)^***^ | 2.015 (1.598-2.540)^***^ | 3.232 (2.551-4.093)^***^ |  |
| Multivarialbe-adjusted^†††^ | 1.000 (Ref) | 2.496 (2.053-3.035)^***^ | 1.999 (1.574-2.538)^***^ | 2.528 (1.955-3.268)^***^ |  |
| Cardiac events†† |  |  |  |  |  |
| Case, n(%) | 568 (13.9%) | 649 (24.2%) | 304 (21.1%) | 300 (31.2%) | 1821 (19.8%) |
| Unadjusted | 1.000 (Ref) | 1.987 (1.753-2.252)^***^ | 1.665 (1.426-1.943)^***^ | 2.816 (2.394-3.314)^***^ |  |
| Age and sex-adjusted | 1.000 (Ref) | 1.855 (1.632-2.108)^***^ | 1.551 (1.327-1.812)^***^ | 2.431 (2.056-2.875)^***^ |  |
| Multivarialbe-adjusted^†††^ | 1.000 (Ref) | 1.630 (1.421-1.870)^***^ | 1.454 (1.235-1.712)^***^ | 1.970 (1.642-2.365)^***^ |  |

† CVD including stroke and cardiac events.

†† Cardiac events included myocardial infarction, coronary heart disease, angina, congestive heart failure, or other heart problems.

^†††^ Multivarialbe-adjusted for age, gender, education, marital status, residence, BMI, drinking, smoking, and chronic comorbidities.

^*^p<0.05; ^**^p<0.01; ^***^p<0.001.
