# Supplementary material for: Independent and joint associations of hypertension and depression with cardiovascular diseases and all-cause mortality: a population-based cohort study
Source: J Hum Hypertens. 2025 Jul 17;39(9):634–42. doi: 10.1038/s41371-025-01045-1 (PMC12417204; doi:10.1038/s41371-025-01045-1)
Supplement: Supplementary file 2 — Supplementary material 2 [file 41371_2025_1045_MOESM2_ESM.docx]

**Supplementary table 2. Sensitivity analyses with further adjustment for** **antidepressant medications.**

| Variables | Neither hypertension nor depression | Hypertension alone | Depression alone | Both hypertension and depression |
| --- | --- | --- | --- | --- |
| All-cause mortality |  |  |  |  |
| Multivarialbe-adjusted^†††^ | 1.000 (Ref) | 1.424 (1.143-1.776)^**^ | 1.032 (0.802-1.328) | 1.547 (1.199-1.996)^**^ |
| CVD† |  |  |  |  |
| Multivarialbe-adjusted^†††^ | 1.000 (Ref) | 2.039 (1.786-2.327)^***^ | 1.595 (1.386-1.835)^***^ | 2.314 (1.978-2.706)^***^ |
| Stroke |  |  |  |  |
| Multivarialbe-adjusted^†††^ | 1.000 (Ref) | 2.790 (2.257-3.448)^***^ | 2.071 (1.642-2.613)^***^ | 2.944 (2.307-3.756)^***^ |
| Cardiac events†† |  |  |  |  |
| Multivarialbe-adjusted^†††^ | 1.000 (Ref) | 1.702 (1.471-1.970)^***^ | 1.428 (1.224-1.666)^***^ | 2.106 (1.779-2.493)^***^ |

^†††^ Multivarialbe-adjusted for age, gender, education, marital status, residence, BMI, drinking, smoking, antidepressant medications and chronic comorbidities.

^*^p<0.05; ^**^p<0.01; ^***^p<0.001.
